# Supplementary material for: Inflammatory Profile Assessment in a Highly Selected Athyreotic Population Undergoing Controlled and Standardized Hypothyroidism
Source: Biomedicines. 2024 Jan 22;12(1):239. doi: 10.3390/biomedicines12010239 (PMC10813236; doi:10.3390/biomedicines12010239)
Supplement: Supplementary file 1 [file biomedicines-12-00239-s001.zip › biomedicines-2810304-supplementary.pdf]

Mann Whitney U-test		P
WBC_sosp	1621	0.029
PLT_sosp	1639	0.025
Neu_sosp	1486	0.005
Linf_sosp	1997	0.623
Mon_sosp	1527	0.007
Eosinofili_sosp	1679	0.060
Basofili_sosp	1937	0.357

Table S1. Mann Whitney U-test of White blood cells and Platelets according to sex in total sample.

Mann Whitney U-test		P
NLR	1361	< .001
PLR	1963	0.519
MLR	1269	< .001
SII	1711	0.077
SIRI	1229	< .001
AISI	1478	0.005

Table S2. Mann Whitney U-test of BIIXs according to sex in total sample.

Kruskal-Wallis

	χ^2	Gdl	P	ε^2
NLR	6.76	2	0.034	0.0683
PLR	1.64	2	0.440	0.0166
MLR	3.95	2	0.139	0.0399
SII	8.38	2	0.015	0.0846
SIRI	11.62	2	0.003	0.1173
AISI	10.55	2	0.005	0.1066

Table S3. Kruskal-Wallis test of BIIXs according to BMI

		Age	TSH	FT4	FT3
NLR	p-value	0.150	0.93	0.32	0.20
PLR	p-value	0.623	0.33	0.37	0.50
MLR	p-value	0.485	0.87	0.06	0.06
SII	p-value	0.298	0.25	0.96	0.72
SIRI	p-value	0.212	0.70	0.13	0.06
AISI	p-value	0.349	0.27	0.46	0.10

Table S4. Correlation of BIIXs with age, TSH, FT4, and FT3 in total sample.

Mann Whitney U-test		P
NLR	1079	0.005
PLR	1214	0.037
MLR	936	< .001
SII	1235	0.051
SIRI	1169	0.020
AISI	1285	0.084

Table S5 Mann Whitney U-test of BIIXs according to Thyroglobulin level in total sample.
